# Isotemporal Substitution Effect of 24-Hour Movement Behaviors on Well-Being, Cognition, and BMI Among Older Adults

**DOI:** 10.3390/jcm14030965

**Published:** 2025-02-03

**Authors:** John Oginni, Suryeon Ryu, Yingying Chen, Zan Gao

**Affiliations:** 1 Department of Kinesiology, Recreation, and Sport Studies, The University of Tennessee, 1914 Andy Holt Avenue, Knoxville, TN 37996, USA; joginni@vols.utk.edu (J.O.); sryu6@utk.edu (S.R.); 2 Edson College of Nursing and Health Innovation, Arizona State University, 500 North 3rd Street, Phoenix, AZ 85004, USA; yingying.chen.6@asu.edu

**Keywords:** cognition, movement behavior, physical activity, sedentary time, sleep

## Abstract

**Background**: This study investigated the interdependent relationships among older adults’ daily engagement in physical activity (PA), sedentary time (ST), sleep, and their well-being, cognition, and body mass index (BMI). **Method**: Forty healthy older adults (31 females; Mean [age] = 70.8 ± 5.58) were included in the analysis. Participants wore a Fitbit tracker for an average of 23 h a day, five days a week, over six months. The Fitbit device tracked lightly active time, active time, ST, and sleep durations. Quality of life and cognitive flexibility were assessed using validated instruments. BMI was calculated using participants’ self-reported height and weight. A compositional analysis (CODA) investigated the codependent associations among these variables and model time reallocation between behaviors. **Results**: Regression models utilizing CODA indicated significant associations between the outcomes of BMI (*p* = 0.05; Adj. R^2^ = 0.20), while cognitive flexibility and quality of life revealed no association (*p* > 0.05). Shifting 10 min from ST to active time is associated with a theoretical decrease of −0.76 (95% CI, −1.49 to −0.04) units in BMI. Similarly, reallocating 10 min from active time to ST is associated with a theoretical increase of 1.17 (95% CI, 0.03 to 2.3) units in BMI. Reallocating 10 min between other movement behaviors yielded no statistical significance. **Conclusions**: Our study highlights the importance of promoting active time to improve BMI in this population. Encouraging 10 min bouts of PA among older adults, in place of ST, is vital for improving national PA guideline adherence.

## 1. Introduction

Globally, there is a significant trend towards an aging population [1]. The term “population aging” describes the rise in the number and proportion of older adults (aged ≥60 years) and the decline of young people (aged ≤15 years) [2]. In other words, population aging is the shift of the population distribution towards older ages [3]. In 2020, the number of people aged 60 and over surpassed that of children under five for the first time. According to the projection, the percentage of people over 60 will increase from 12% to 22% of the global population [3]. Aging populations lead to some challenges for societies and businesses including slowing economic growth and possible poverty among older adults [4]. As our global society ages rapidly, we potentially face a future where older adults may experience poverty and lack access to care in already overburdened healthcare systems. In this context, achieving healthy aging is fraught with challenges [5]. The process that develops and maintains functional ability enabling well-being is expedient in older populations [5]. At the core of optimal aging are disease and disability prevention, quality of life enhancement, maintenance of good physical and cognitive functionality, and engagement in healthy behaviors and active lifestyles [6]. Data captured through the National Health and Nutrition Examination Survey (NHANES) in individuals aged 65 years and older showed a 1.5- to 1.9-fold increase in the prevalence of grade 1 obesity, defined as body mass index (BMI) 30.0 to 34.9 kg/m^2^, which was observed between 2015 and 2018 compared with 1988 to 1994 [7]. However, physical activity (PA) can have a positive impact [8] on the BMI of older adults, but a decline in BMI in older age can also have negative health consequences [9]. Overall, it is important to avoid excessive body fat and to regularly participate in PA to prevent or slow down the loss of functioning as one ages [10,11,12]. 

A day is made of 24 h which involves a sequence of movements and behaviors. This includes sedentary behaviors (SB), sleep, light PA, and moderate-to-vigorous physical activity (MVPA) [13,14,15]. A change in the amount of time spent in any of the four-movement behaviors will change the amount of time spent in another [16]. While the exploration of compositional data analysis in daily movement behaviors is in its early stages, it sheds light on the connection between the distribution of time across different activities and health outcomes. Few studies have utilized the concept of compositional analysis to investigate 24-hour (24 h) movement behaviors with claims that movement behavior is associated with health outcomes [17] like blood pressure, and anthropometric measures [16,18,19]. However, few studies on older adults’ 24 h movement behaviors and their effect on well-being, most especially cognition, remain scarce. A recent study from Taiwan evaluated the relationship between PA and cognitive function in older adults by replacing 30 min of sedentary behavior or sleep with light PA [20]. However, this study utilized a subjective measure of cognitive function, potentially subjected to recall bias. Also, there might be a potential concern for adherence to a bout of 30 min of MVPA and light PA among older adults. Multiple short bouts of PA might also improve adherence to PA in adults [21], most especially among older adults. A recent longitudinal study [22] of a theoretical reallocation of PA into sedentary behavior, standing, and time in bed revealed an association with a prospective decline in quality of life. To address this gap, our study focused on 10 min time-reallocation of movement behaviors and sleep and the use of objective measures of cognition to eliminate recall bias.

This study aimed to investigate the association of time allocation of 24 h movement behaviors between older adults’ BMI, cognition, and well-being outcomes. We hypothesized that there would be a significant association between the reallocation of at least 10 min among different movement behaviors, BMI, quality of life, and cognition, such that changes in the allocation of time lead to corresponding changes in the outcome variables. This study contributes to the literature by using isometric log-ratio coordinates to maintain the relative differences between components of PA. In addition, objective measures of PA and cognition add to the quality of the data. We anticipate that the findings from this study will provide insights to future researchers and health professionals looking into the compositional and potential bout relationships between human movement behaviors and older adults’ cognitive health, well-being, and BMI. Additionally, this knowledge can aid in developing effective lifestyle interventions and strategies to improve adherence to PA with benefits on health outcomes among older adults.

## 2. Materials and Methods

This cross-sectional study utilized baseline data obtained from the parent study conducted majorly in Minnesota with a few participants from other US states. The protocol was approved on 8 February 2023, by the University of Minnesota’s Institutional Review Board (IRB ID: STUDY00017966). Prior to participation, all participants provided written informed consent.

### 2.1. Study Design and Participants

The parent study which employed a cross-over trial intervention enrolled individuals aged 60 years and above. The recruitment was conducted via emails, social media, and word of mouth through recruitment flyers locally in Minnesota with few participants from other states in the U.S. Eligible and interested individuals were scheduled for screening and enrollment via phone calls or a video call. Inclusion criteria include: (1) aged 60 years or old; (2) do not have cognitive impairment or physical/mental disabilities that might limit the practice of Tai Chi; (3) provide consent; and (4) possess basic English communication capability. Exclusion criteria include: (1) declined completion of the informed consent; (2) diminished capacity to consent; and (3) physical or mental disabilities. The parent study began in March 2023 and concluded by October 2023, with 44 individuals consenting. Of these participants, three had incomplete overall data, while one had incomplete PA data. A total of 40 participants were included in the present analysis.

### 2.2. Measures

Demographic and anthropometric information. Demographic information including age, sex, and race was collected remotely from participants. Likewise, weight and height were self-reported by the participant using a standardized data sheet through Qualtrics online survey [23], a survey platform that can be used to easily administer questionnaires remotely. The BMI of the participants was calculated using the weight (kg) divided by height (m^2^).

Twenty-four-hour movement behaviors. Movement behaviors were assessed using Fitbit tracker (Inspire 3) owned by Google (Mountain View, CA, USA). The Fitbit device tracked sleep data and PA data including steps, activity intensity (lightly active, fairly active, and very active minutes), and energy expenditure [24]. For this study, the participants were instructed to wear the Fitbit tracker on the non-dominant wrist at all times throughout the study except for showers, and deep-water activities. In this study, the researchers retrieved Fitbit data from its Application Programming Interface (API) feature through a third-party company Fitabase (Fitabase LLC, San Diego, CA, USA). PA was tracked and synchronized to its Fitbit application on participants’ mobile devices (e.g., tablets or smartphones) where they were uploaded to the Fitbit server weekly. Fitbit’s proprietary activity categories were defined using metabolic equivalent task [25] (MET) calculations by Jette et al. [26]. Fitbit’s PA intensity classification aligns with the PA guidelines for Americans, which utilizes MET to quantify energy expenditure for different activities [26,27]. One MET represents the energy expended while sitting at rest. Light-intensity activities involve non-sedentary waking behaviors that require less than 3.0 METs, while moderate-intensity (i.e., fairly active) activities require between 3.0 and 6.0 and vigorous-intensity (i.e., very active) activities demand 6.0 or more METs [27]. In this study, lightly active time was used to measure light PA, while fairly active time and very active time were combined to represent MVPA.

Quality of life. This was assessed by the brief Older People’s Quality of Life (OPQOL) questionnaire [28] with Cronbach’s alpha of 0.86. This questionnaire has 13 items. Question prompt includes ”I enjoy my life overall, I look forward to things, I feel safe where I live, and I have enough money to pay for household bills”. Each of the 13 items is scored on a 5-point Likert scale. The items were summed for a total OPQOL-Brief score. Questionnaires were administered remotely to all participants.

Cognitive flexibility. This was assessed remotely and objectively via the digital card-sorting game (EFgo™). To facilitate remote assessment, the online version (https://reflectionsciences.com/measure/ (accessed on 13 January 2025) [29] of the cognitive test, which is similar to the digital card sorting game by the National Institutes of Health Toolbox, was used. The application provides both visual and audio instructions on how to navigate the test exercise. Participants were asked to match a series of picture pairs to a target picture and switch between tasks as assigned by the application. The final score of each participant was sent to the database and retrieved by a research assistant.

### 2.3. Statistical Analysis

The data analysis was conducted in R version 4.3.1 using the packages ”composition” and ”Codaredistlm” for compositional data analysis. For data analysis, the research assistant combined fairly active and very active minutes into a single category named active time, equivalent to MVPA minutes, a variable commonly used in research. One week of movement behavior (lightly active time, active time, ST, and sleep time) data was used for analysis. Descriptive statistics of mean, standard deviation, and percentage were utilized to characterize demographic characteristics.

Compositional data, representing the average daily time spent in ST, sleep, light active time, and active time, were transformed into isometric log-ratio (ilr) coordinates using the default isometric log-ratio transformation provided by the compositions package. The ilr is one of the different types of log-ratio transformations that captures time spent in all four behaviors [16,30,31,32]. To describe the central tendency of the 24 h movement behaviors, the mean was adjusted to sum to 1440 min (24 h) by calculating the geometric mean of each component, following methodologies described in previous studies [16]. This adjustment addresses the constant sum constraint, which is a common challenge in compositional data analysis. By transforming the data into ilr coordinates, standard statistical analysis techniques can be applied more reliably and effectively. Geometric means and a variation matrix were computed to characterize the composition of movement behaviors, providing valuable insights into the central tendency and variability within the compositional data. Movement behaviors represented as isometric log-ratio coordinates served as explanatory variables in the linear models. Covariates, which included education status, sex, and race, were also included in the model as explanatory variables. The outcome variables were assessed for normality. BMI outcome underwent a natural log transformation while quality of life and cognitive flexibility did not conform to normality assumption even after log transformation (online Supplementary Figures S1 and S2). This necessitated the use of a generalized linear model [33] for cognitive and quality of life outcomes. Untransformed outcomes of BMI, quality of life, and cognitive flexibility were used for time-reallocation between movement behaviors for ease of interpretation and to communicate findings more efficiently. Additionally, linearity was evaluated in the isometric log-ratio multiple linear regression models.

The associations between BMI, quality of life, cognitive flexibility, and movement behaviors (in isometric log-ratio coordinates) were investigated using multiple linear regression models and a generalized linear model (GLM). Subsequently, predictions were generated for new compositions, which were also expressed as isometric log-ratio coordinates. These compositions involved reallocating fixed durations of time from one movement behavior to another while maintaining the remaining behaviors constant. Predictions were computed with reallocations of 10 min. A 10 min increment in activity is recognized to yield positive health benefits according to previous studies [16]. 

## 3. Results

The study participants had an average age of 70.8 years (SD = 5.58). Of the participants, 22.5% identified as male and 77.5% as female. A total of 17.5% were identified as Asian American, 5% as Black or African American, and the majority (77.5%) as White.

Participants wore the Fitbit tracker for an average of 23 h a day, five days a week. Analysis of compositional means for activity behaviors across the sample revealed distinct patterns. Specifically, participants spent an average of 1.6% of their day in active time, 51.78% in ST, 13.68% in lightly active time, and 32.9% in sleep (Table 1). This composition indicated a predominance of sedentary time over active periods, with both lightly active time and active time being lower.

Variability in these behaviors was detailed in the compositional variation matrix (Table 2), indicating the degree of dependence between different activity levels. Near-zero values indicated a high dependency between the compared behaviors. Notably, active time exhibited the highest log-ratio variance, suggesting that the time spent in active time was less dependent on the time spent in other activities. This indicates that the time an individual allocates to active time does not significantly influence the time they spend on other activities.

**Figure 1:**
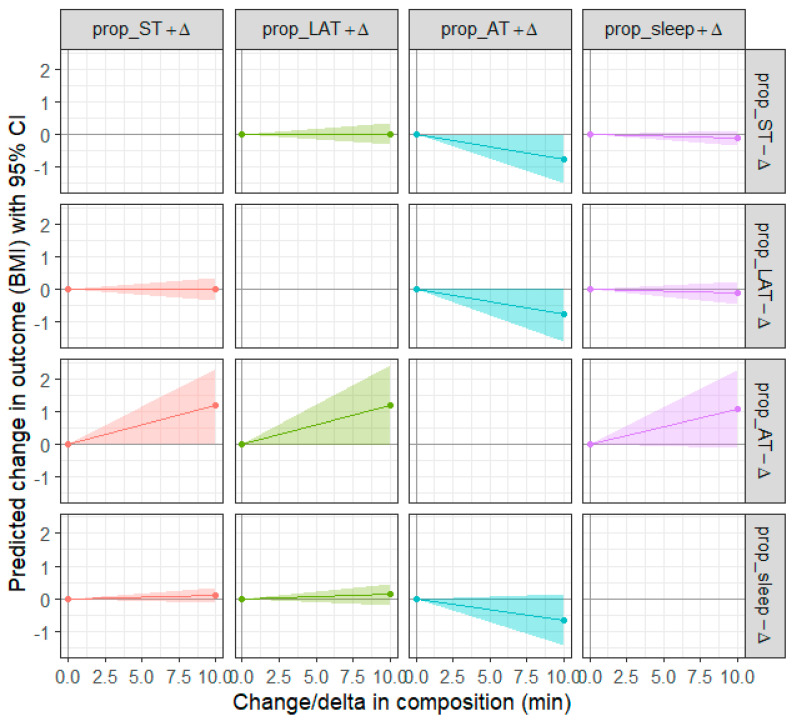
Predicted change in BMI. −∆ = time (10 min) reallocated away from a movement behavior; +∆ = time (10 min) reallocated to a movement behavior. The movement behaviors are Active time (AT), Sedentary time (ST), Lightly active time (LAT), Sleep.

The multiple linear regression model demonstrated a significant association between isometric log-ratio coordinates (ST, active time, lightly active time, sleep) and outcomes of BMI (*p* = 0.05; Adj. R^2^ = 0.20) adjusting for education status, race, and sex. The generalized linear model demonstrated no significant association between isometric log-ratio coordinates and cognitive flexibility (*p* > 0.05) and quality of life (*p* > 0.05). After adjusting for education status, race, and sex, shifting 10 min from ST to active time is associated with a theoretical decrease of −0.76 (95% CI, −1.49 to −0.04) units in BMI (Figure 1). Similarly, moving 10 min from active time to ST is associated with a theoretical increase of 1.17 (95% CI, 0.03 to 2.3) units in BMI. Reallocating 10 min between other movement behaviors yielded no statistical significance.

## 4. Discussion

This study investigated how time reallocation within a 24 h period of movement behaviors impacts BMI, cognitive flexibility, and quality of life in older adults, utilizing compositional data analysis. By utilizing objective measures of 24 h movement behaviors and cognitive flexibility, this study contributes to the expanding research on the compositional nature of 24 h movement behaviors, involving active time, lightly active time, ST, and sleep. In addition, our study focused on the 10 min time reallocation of movement behaviors and sleep on the basis that multiple short bouts of PA might also improve adherence to PA in older adults [21].

Our findings indicated that a significant portion of time for the older adults in our study was spent in ST (51.78%), and the least amount of time was spent on active time (1.6%). This supports a previous study that noted older adults were more likely to have low levels of PA with varying intensity [14,34]. The statistical analysis via a multiple linear regression model highlighted that the time distribution among different activities was significantly associated with BMI. This indicates that how time is allocated among various activities has a significant impact on BMI [35,36]. 

In our study, active time emerged as a key aspect of intervention targeting older adults’ BMI. This finding is supported by previous studies presenting that participating in active time may be beneficial for maintaining a healthy BMI during late adulthood [9]. Consistent with previous studies, our findings showed active time’s dominant effect on BMI when time was reallocated from ST [37,38,39]. Therefore, this study further contributes to the scientific evidence that active time (i.e., MVPA) is a major component of movement behavior that should be considered for healthy body composition regardless of the age group.

A small amount of PA is better than none. In our study, we used 10 min of time reallocation, which is within the range of a minimal bout of PA that can elicit changes in health outcomes [32,40]. Reallocating 10 min to active time might foster adherence to PA for a long time [41], which tends to be suitable for the older population. Adherence to PA is important to see a long-term effect on health outcomes [42], while 10 min of time reallocation might improve adherence to PA and impact body composition positively. In contrast to our studies that revealed no association between movement behaviors, cognition, and quality of life, some studies revealed an association between time reallocation of movement behavior, cognitive flexibility, and quality of life [43,44]. This might potentially be a result of the small sample size of this study. Although studies suggesting an association between movement behaviors, cognitive function and quality of life are still scarce. A longitudinal study by Palmberg et al. [22] revealed a theoretical reallocation of PA into sedentary behavior, standing, and time in bed and found an association with a prospective decline in quality of life, which emphasizes the positive role of PA in place of sedentary activities in promoting better quality of life among the older population. However, there is still a clear need for longitudinal studies to discern the effect of time reallocation between movement behavior, cognition, and quality of life.

The strength of this study includes the utilization of objective measures of 24 h movement behaviors compared to studies that rely on self-reported PA surveys. This approach enables a detailed analysis that considers the collinearity and interdependence of movement behaviors within the constrained timeframe of a single day. In addition, the objective measurement of cognitive flexibility eliminates recall bias, enhancing the quality of the data. This study also utilized a robust regression model to resolve the normality issue for cognitive flexibility and quality of life outcome. However, the limitations of this study include small sample size and normality assumptions not ascertained for cognitive flexibility and well-being outcomes which necessitated the use of GLM. Additionally, Latino American and Black or African American participants were under-represented in the sample. As a result, caution should be exercised when generalizing the findings across these racial groups. This study’s cross-sectional nature primarily reflects variations in the theoretical time allocation for different movement behaviors within the sample rather than actual differences between individuals, which poses challenges to establishing causality [45]. The Fitbit’s accelerometer cut-points for the PA, ST, and sleep are closed source. Likewise, the algorithms tend to overestimate both light-intensity PA and MVPA [46] compared with ActiGraph. Human sleep is a complex multidimensional phenomenon and classifying aspects of sleep is challenging [47]. For instance, sleep duration was the only sleep metric used in this analysis, other metrics such as sleep efficiency and wake after sleep onset might provide more clarity in the association with selected health outcomes. Therefore, caution should be exercised when interpreting the results, particularly regarding the projected changes in BMI. 

## 5. Conclusions

Despite some of the limitations, our study provided key findings that emphasize the promotion of MVPA (i.e., active time) to improve BMI for healthy body composition in this older population. The promotion of a 10-minute bout of MVPA among the older population is vital for improving PA adherence. For future studies, researchers are encouraged to utilize a larger sample size for follow-up studies to reduce concerns about normality and incorporate longitudinal prospective designs to observe changes, identify patterns, and establish temporal relationships that suggest a cause-and-effect relationship [48].

## Figures and Tables

**Table 1:** Baseline characteristics.

	Overall (N = 40)
Age (years)	70.8 (5.58)
Sex	
Male	22.5%
Female	77.5%
Race	
Asian American	17.5%
Black or African American	5%
White	77.5%
Education	
College Graduate	30%
Graduate School	57.5%
Some college or technical training school	10%
High School	2.5%
Compositional mean	
Active time	1.6%
ST	51.78%
Lightly active time	13.68%
Sleep	32.9%

ST = Sedentary time. Data are presented as mean ± SD for continuous variables and percentage for race, sex, and education being a categorical variable.

**Table 2:** Compositional variation matrix.

	Active Time	ST	Lightly Active Time	Sleep
Active time	0	1.09	1.01	1.02
Sedentary Time	1.09	0	0.16	0.08
Lightly active time	1.01	0.16	0	0.15
Sleep	1.02	0.08	0.15	0

## Data Availability

The datasets used and/or analyzed during the current study are available from the corresponding author upon reasonable request.

## Supplementary Materials

The following supporting information can be downloaded at: https://www.mdpi.com/article/10.3390/jcm14030965/s1, Figure S1: Histogram of Cognitive Flexibility; Figure S2: Histogram of Quality of Life.
