# Supplementary figures and images for: Isotemporal Substitution Effect of 24-Hour Movement Behaviors on Well-Being, Cognition, and BMI Among Older Adults
Source: J Clin Med. 2025 Feb 3;14(3):965. doi: 10.3390/jcm14030965 (PMC11818513; doi:10.3390/jcm14030965)

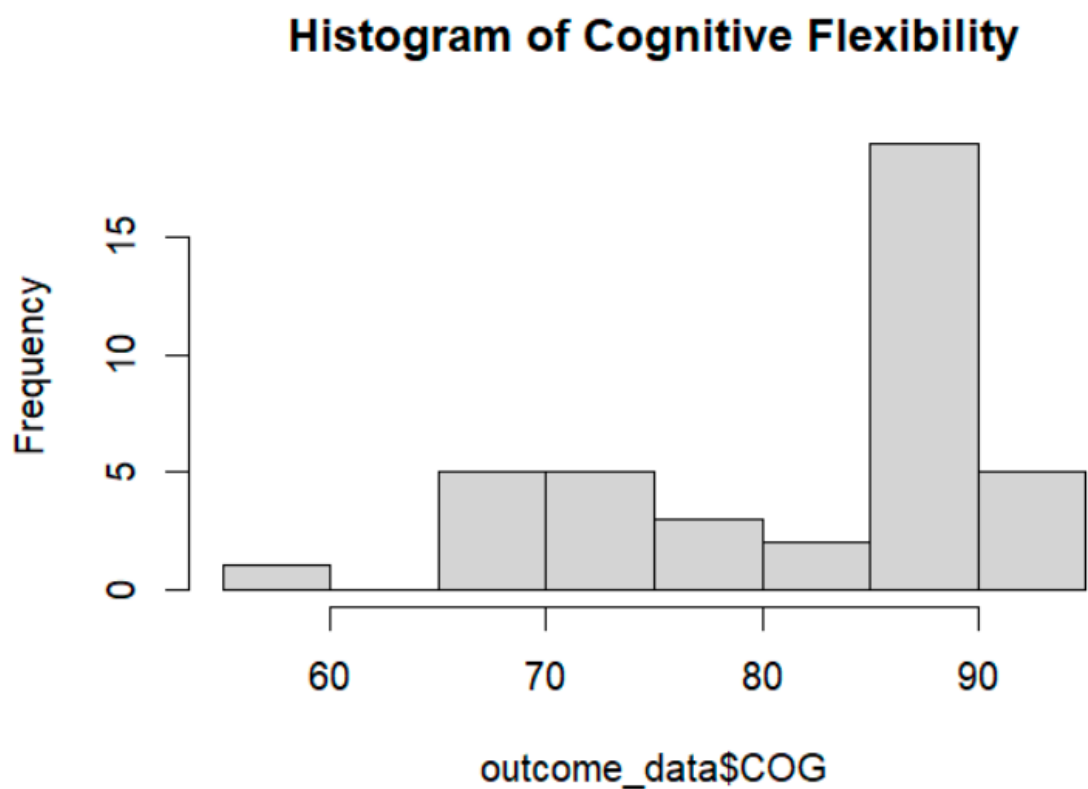

Figure S1: Histogram of Cognitive Flexibility.

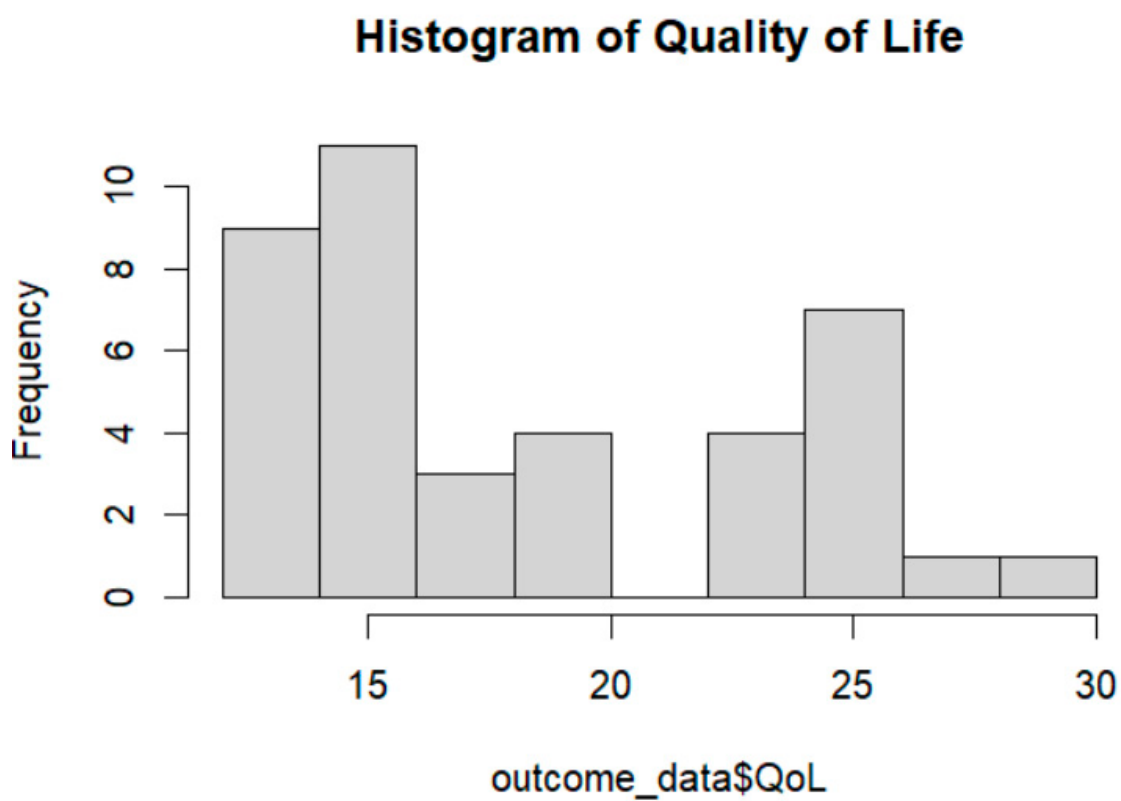

Figure S2: Histogram of Quality of Life.

Supplement: Supplementary file 1 [file jcm-14-00965-s001.zip › jcm-3401670-supplementary.pdf]
